# Supplementary material for: De novo mutations implicate novel genes in systemic lupus erythematosus
Source: Hum Mol Genet. 2017 Nov 21;27(3):421–9. doi: 10.1093/hmg/ddx407 (PMC5886157; doi:10.1093/hmg/ddx407)
Supplement: Supplementary Tables [file supplementary_material_ddx407.pdf]

Supplementary Material

Supplemental Data include seven figures and six tables.

Web Resources

The URLs for data presented herein are as follows:

1000 Genomes Project, http://www.1000genomes.org/
Exome Aggregate Consortium (ExAC), http://exac.broadinstitute.org/
NHLBI Exome Variant Server (ESP), http://evs.gs.washington. edu/EVS
Online Mendelian Inheritance in Man (OMIM), http://www.omim.org/.
RefSeq, http://www.ncbi.nlm.nih.gov/refseq/

Phyre2, http://www.sbg.bio.ic.ac.uk/phyre2/html/page.cgi?id=index

Supplementary Methods

Sequencing and alignment. Sequencing libraries were prepared from 1ug of DNA using the SureSelect XT Human All exon v4 +UTR kit (Agilent Technologies). The libraries were prepared according to the manufacturer's manual (SureSelect XT target enrichment system for Illumina paired end sequencing library, v1.4.1 Sept2012).The exome capture libraries from each individual in the trio were sequenced on Illumina HiSeq 2000 as 100bp paired-end reads. The resulting BCL files from the sequencer were processed with Illumina Casava software v1.8 to obtain paired end reads in FastQ format. The paired end FastQ reads were aligned to the human reference genome hg19 (GCRh37) using Novoalign v2.07.11 with the following parameters (-i 200 30 -o SAM -o SoftClip -k -a -g 65 -x 7). The resulting BAM file was processed to sort and remove PCR duplicates using Picard tools. Only reads uniquely aligned to the reference genome were considered for further analysis. At the end of this process one BAM file per individual was obtained totaling 90 BAM files (30 trios).

Quality control (QC) and variant annotation. Variants were retained if they passed the following criteria: (i) read depth ≥20x, (ii) located within exome-captured regions as annotated in Gencode (on-target) and NCBI RefSeq annotation(36). Read depth estimation was performed using DepthOfCoverage from GATK tool(37), as evaluated using bedtools(38). All but one family had 75% representation of the exome at 20X (Fig. S1). Variants passing these QC criteria were annotated using ANNOVAR(39). Any variant observed in either ExAC, 1000 genomes, ESP6500, or in-house databases was considered polymorphic.

DNM calling

Three bioinformatics tools previously been used in DNM identification studies (36, 37) were used for DNM screening: BCFtools (38), DeNovoGear (39) and DeNovoCheck (36). For each identified DNM, BCFtools assigns a combined likelihood ratio score (CLR, range 1-255) and DeNovoGear assigns a posterior probability score (PP_dnm, range 0.0 – 1.0), while DeNovoCheck flags a variant as 'denovo' without providing a score. Conservative threshold scores for ascertainment of DNM were applied: CLR ≥ 80 for BCFtools and PP_dnm ≥ 0.8 for DeNovoGear (Fig. S5). 454 variants were identified at these thresholds. Eight additional variants that were identified by DeNovoCheck and validated by IGV, resulting in a total of 462 variants, which map to 257 genes. The variants were next filtered sequentially filtered (Fig. S2): (A) Removal of NGS error prone genes (NEPG): genes previously reported as probable false positive signals from NGS studies due to high frequency of rearrangements, polymorphisms or present in multiple copies(40); (B) Fulfil a *de novo* pattern of inheritance: any variant that supports Het:Ref:Ref for Child:Father:Mother, respectively, was considered a potential DNM. We also further selected variants that did not contain any trace of alternate allele in any of the parents. IGVtools was used to count the number of non-reference bases at each identified variant position from both father and mother. Only variants with a zero count of non-reference bases in both father and mother were considered as very high quality variants and retained for further analysis; (C) Variant annotation: only non-silent variants

(Missense, Nonsense, splicing and insertions/deletions) was retained for further analysis. This process resulted in a total of 17 variants in 17 genes (Table S1).

Analysis of whole exome sequencing (WES) in cases only. To quantify the advantage of using parent-offspring trios, the whole exome samples of the 30 probands were analysed alone. 584,798 variants with ≥20X coverage depth and within Gencode capture regions were identified. All stringent filters to help in refinement of variants that could be potentially causal were applied. These include two filters previously described in the *de novo* filtering approach (Non-NEPG and variant annotation) along with selection of heterozygous variants (based on the pattern of zygosity expected for DNM) and non-polymorphic filters (variants not observed in control datasets). To note, this is unlike the trio analysis where variants were not filtered for non-polymorphic. Filters were applied sequentially 1) Non-NEPG 2) Variant annotation 3) Heterozygosity 4) Non-polymorphic and resulted in 1194 variants in 1067 genes (Fig.S3).

Figure S1. Exome Capture

Percentage of the exome captured at different depths in all three samples of each family trio. The x-axis represents sample name of the case in a trio. At each given depth all the 3 samples in the trio pass the threshold.

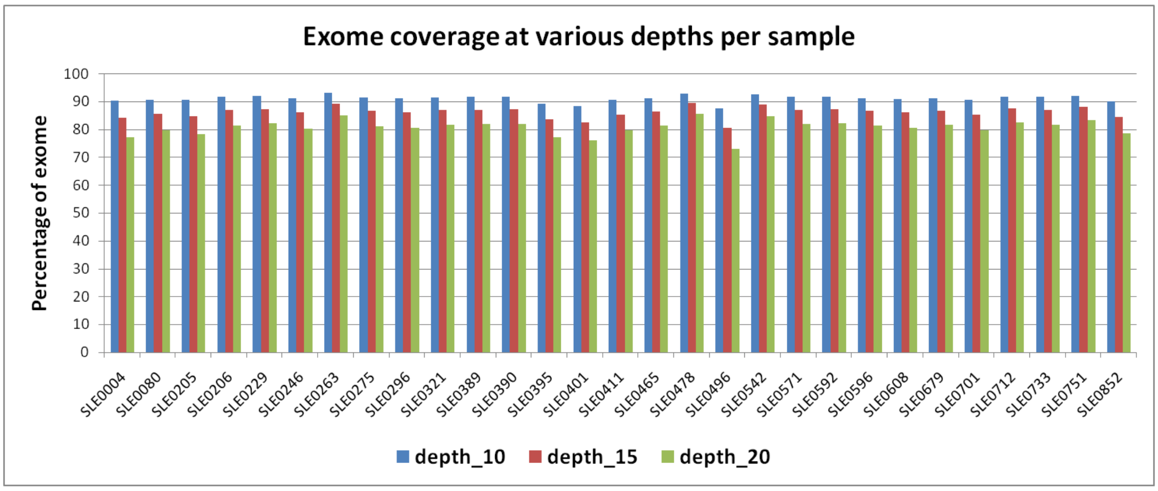

Figure S2. Variant filtering to identify putative *de novo* mutations

(A) Overlap of mutation identified with three bioinformatic tools and subsequent sequential filtering. B) Number of variants present in each SLE proband at each step of filtering process. V= variants; g=genes.

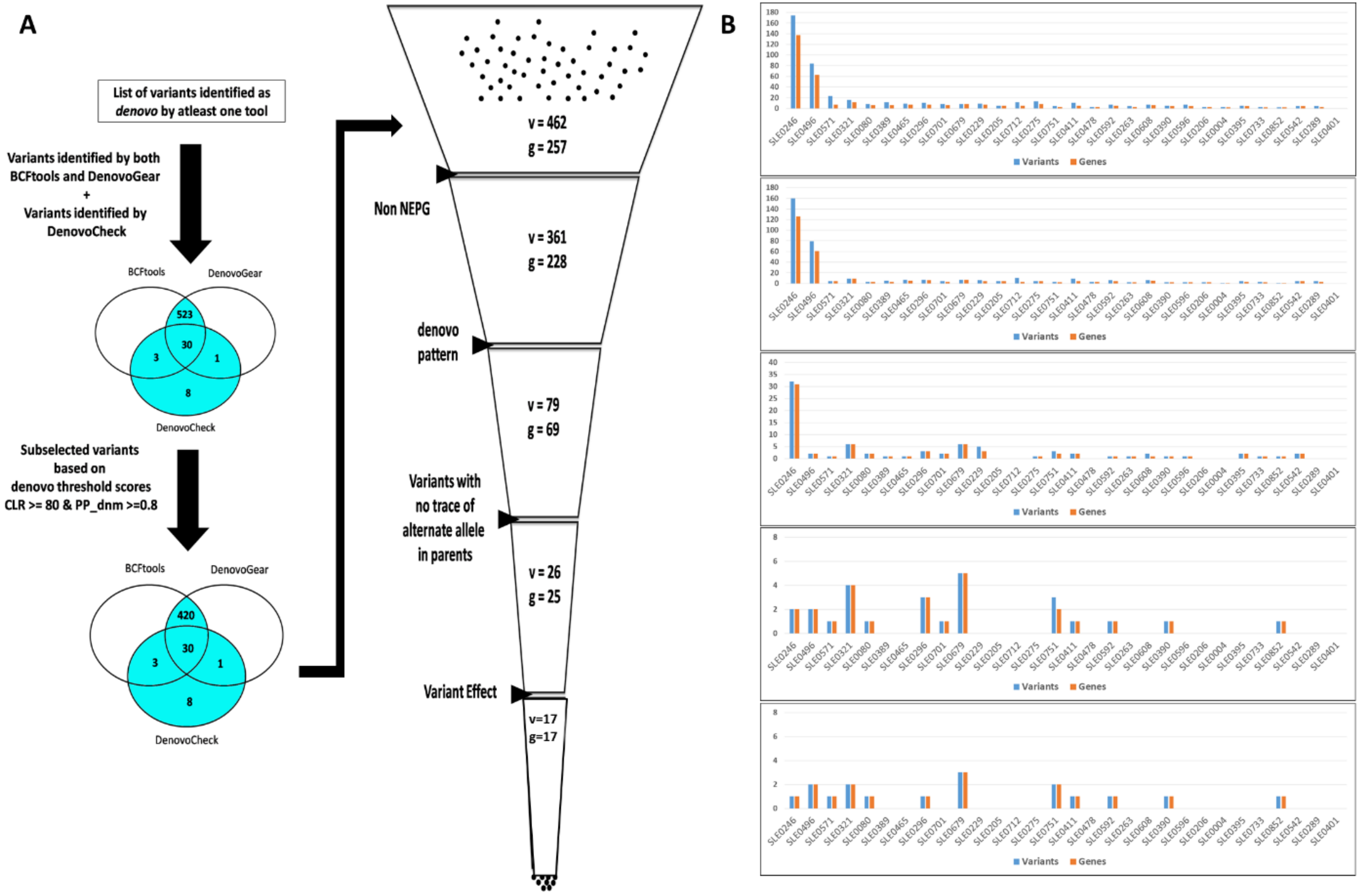

Figure S3. Case-only analysis of WES

A) Sequential filtering of variants B) Overlap of variants identified in case-only and trio analyses. V= variants; g=genes.

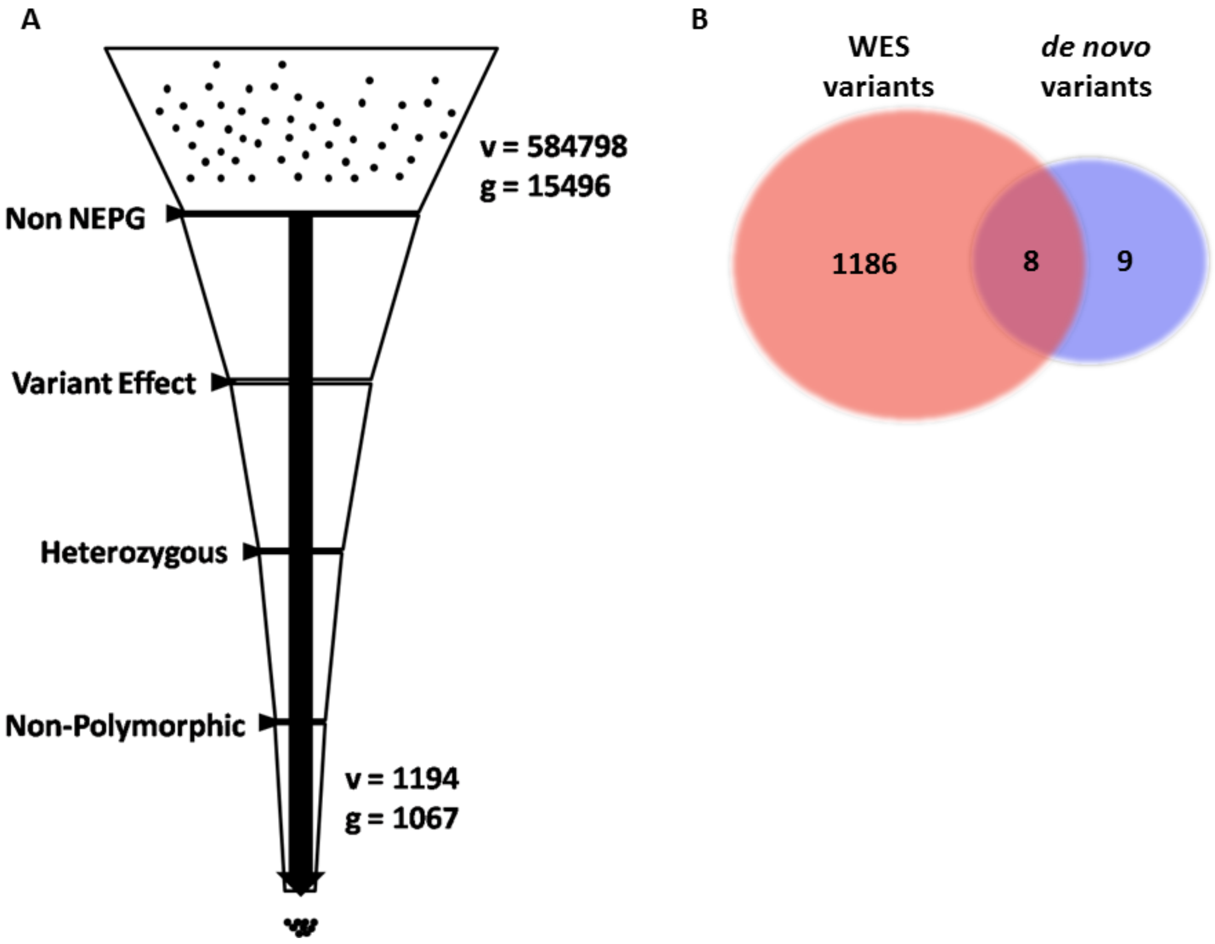

Figure S4. Expression profiles of genes with *de novo* mutations

Expression data for each of the *de novo* mutation genes across all cell/tissue types were downloaded from BioGPS and $\log_2$ normalized. A) Expression data across all cell types are displayed, with a mean expression for tissues with multiple cell types B) Expression data for 14 immune cell/tissue types are listed. * donates cell/tissue with highest expression for given gene. *PNPLA1* and *UBN2* have uniform expression across all cells/tissues. No data are available for *HMSD*.

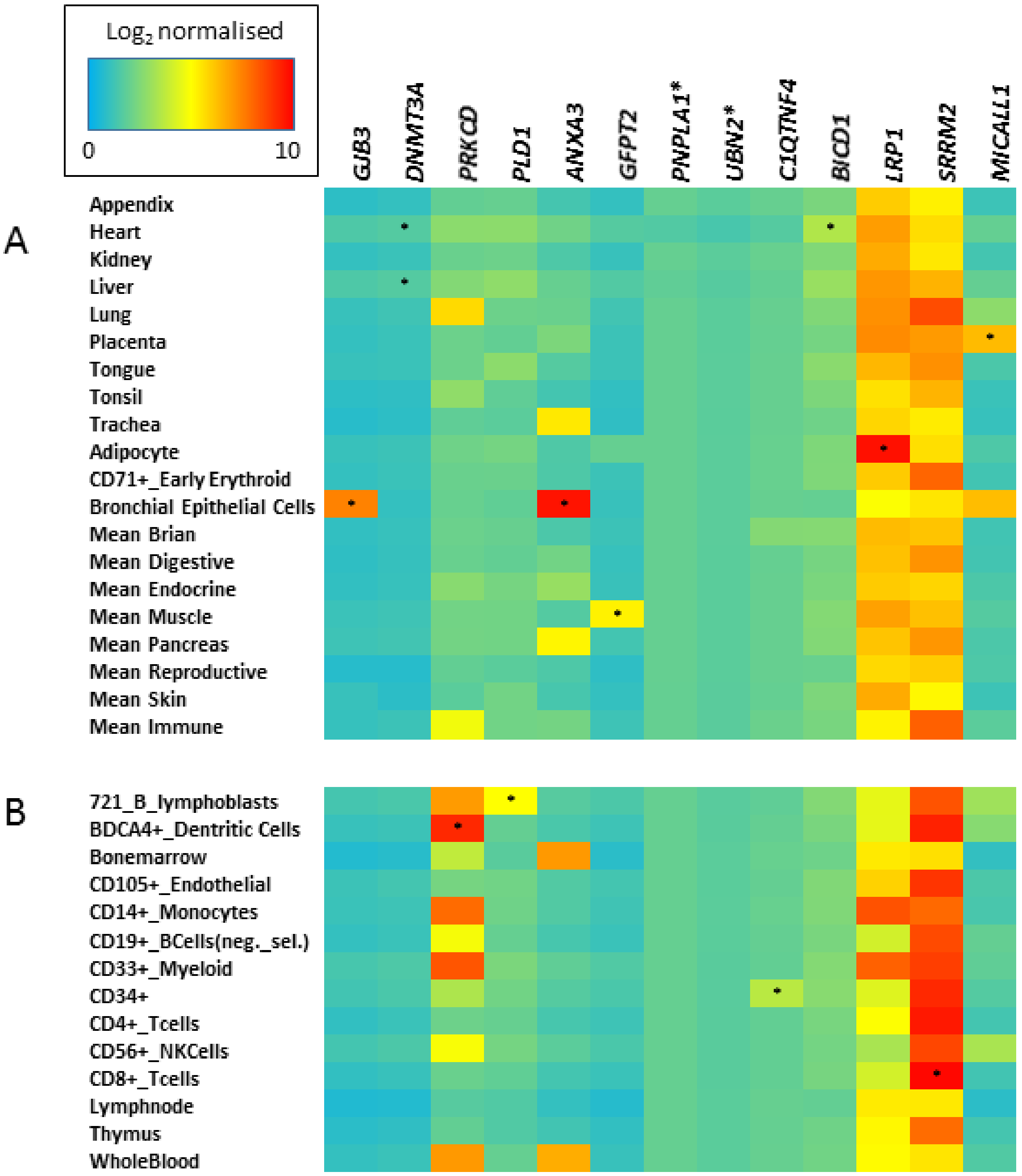

Figure S5. Threshold calculations for BCFtools and DenovoGear.

Calculating the threshold scores from BCFtools (x-axis) and DenovoGear (y-axis) to maximize the number of variants identified by the two tools. The intersection of the horizontal and vertical lines represent the calculated threshold scores for CLR and PP_dnm (≥80 CLR and > 0.8 PP_dnm scores). The cluster to the left represents variants identified by DenovoGear only and their respective PP_dnm scores. The cluster on the bottom represents variants identified by BCFtools only and their respective CLR scores.

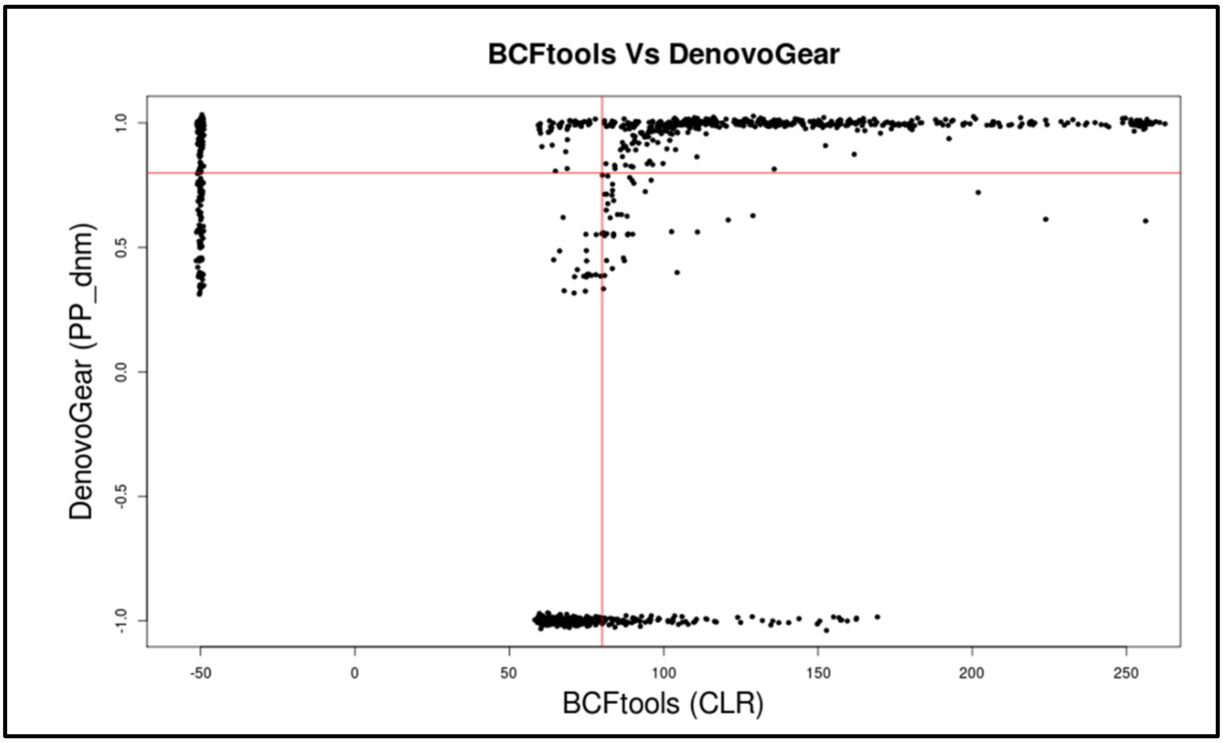

Figure S6. Info Score threshold testing through genotype concordance

Concordance of genotype calls from WES and imputed data from SLE cases (n=30) across increasing info score threshold (0.2-0.8) as tested using Plinkv1.9. Concordance of genotype calls from WES and variants directly typed on OmniBead 1 is shown as reference. Info score threshold of 0.3 was used in further analyses.

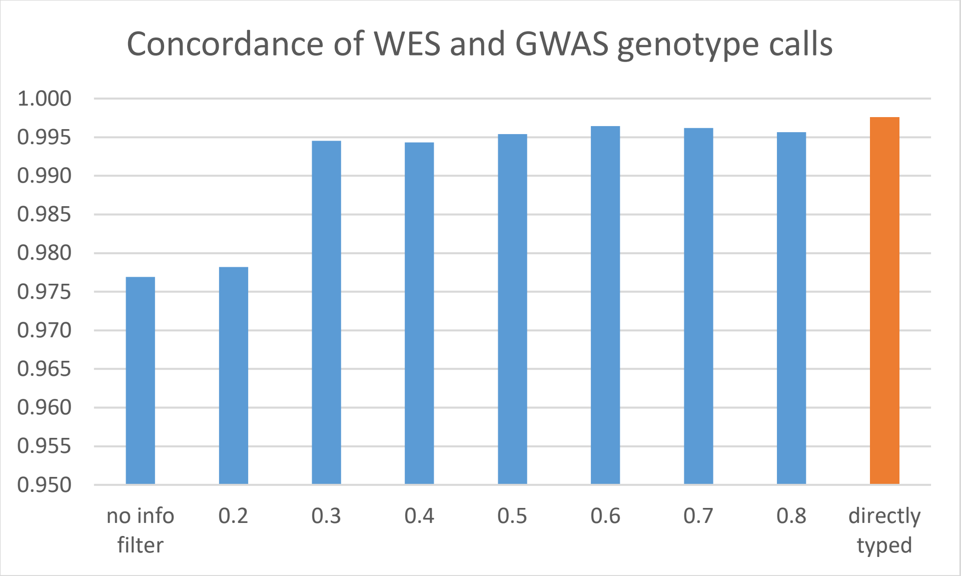

Figure S7. Distribution of info scores from IMPUTE for rare (<1%) variants (n=446) used in burden testing.

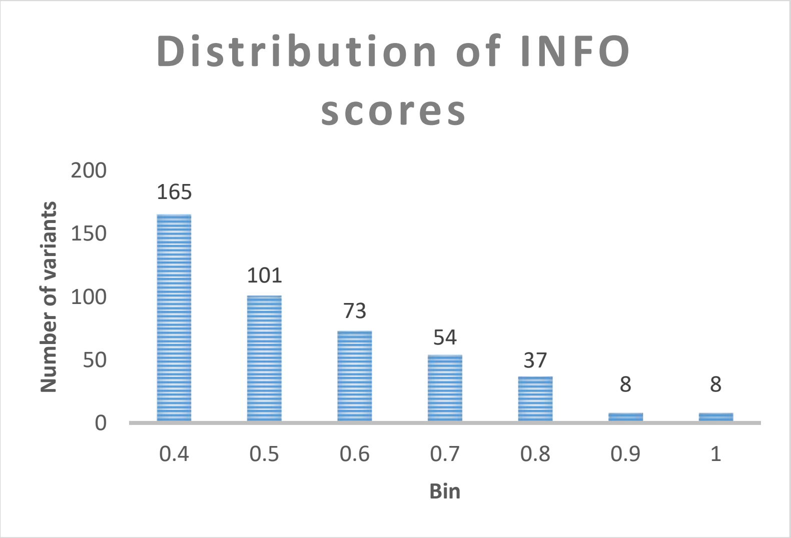

Table S1. Summary of putative *de novo* mutations identified through trio analysis of WES

Family	Chr	Position	Ref	Alt	Gene	Gene Description	Exon	Base	Amino acid	Tools[a]
SLE0321	1	35251125	C	G	*GJB3*	gap junction protein, beta 3, 31kDa	2	762C>G	Asp254Glu	B, DG, DC
SLE0296	2	25457236	G	A	*DNMT3A*	DNA (cytosine-5-)-methyltransferase 3 alpha	19	2084C>T	Ala695Val	B, DG, DC
SLE0496	3	53223122	G	A	*PRKCD*	protein kinase C, delta	16	1603G>A	Gly535Arg	B, DG, DC
SLE0571	4	79512728	G	T	*ANXA3*	annexin A3	7	434G>T	Ser145Ile	B, DG, DC
SLE0411	5	179743769	C	T	*GFPT2*	glutamine-fructose-6-phosphate transaminase 2	12	1147G>A	Val383Met	B, DG, DC
SLE0679	7	138968784	C	A	*UBN2*	ubinuclein 2	15	3133C>A	Phe1045Thr	B, DG, DC
SLE0852	11	47611769	G	C	*C1QTNF4*	C1q and tumor necrosis factor related protein 4	2	594C>G	His198Gln	B, DG, DC
SLE0390	12	32369376	G	C	*BICD1*	bicaudal D homolog 1 (Drosophila)	2	409G>C	Val137Leu	DC
SLE0679	12	57588368	C	T	*LRP1*	low density lipoprotein receptor-related protein 1	50	8077C>T	Arg2693Cys	B, DG, DC
SLE0080	16	2812426	C	T	*SRRM2*	serine/arginine repetitive matrix 2	11	1897C>T	Arg633Cys	B, DG, DC
SLE0321	18	61621642	G	A	*HMSD*	histocompatibility (minor) serpin domain containing	3	73G>A	Ala25Thr	DC
SLE0751	21	45971082	G	A	**KRTAP10-2*	keratin associated protein 10-2	1	260C>T	Phe87Leu	DC
SLE0246	1	183205708	G	C	**LAMC2*	laminin, gamma 2	17	2570G>C	Arg857Phe	DC
SLE0679	3	171431716	G	A	*PLD1*	phospholipase D1, phosphatidylcholine-specific	9	878C>T	Thr293Met	B, DG, DC
SLE0592	6	36260896	G	A	*PNPLA1*	patatin-like phospholipase domain containing 1	3	497G>A	Arg166His	B, DG, DC
SLE0679	12	10599177	T	C	**KLRC1*	killer cell lectin-like receptor subfamily C, 1	7	675A>G	Ile225Met	B, DG, DC
SLE0751	22	38336799	C	T	*MICALL1*	MICAL-like 1	16	2554C>T	Arg852Cys	DC

*mutations not confirmed by Sanger Sequencing

[a] B=BCFtools, DC=DeNovoCheck DG=DenovoGear

Table S2. Family pedigrees used in Sanger Sequencing confirmation of de novo variants

Family	Parents	Affected Proband	Unaffected Sibling
SLE0080	SLE0080_1-1<br>SLE0080_1-2	SLE0080_2-1	-
SLE0296	SLE0296_1-1<br>SLE0296_1-2	SLE0296_2-1	SLE0296_2-2
SLE0321	SLE0321_1-1<br>SLE0321_1-2	SLE0321_2-1	SLE0321_2-2
SLE0390	SLE0390_1-1<br>SLE0390_1-2	SLE0390_2-2	SLE0390_2-1
SLE0411	SLE0411_1-1<br>SLE0411_1-2	SLE0411_2-2	SLE0411_2-1
SLE0496	SLE0496_1-1<br>SLE0496_1-2	SLE0496_2-1	SLE0496_2-2
SLE0571	SLE0571_1-1<br>SLE0571_1-2	SLE0571_2-3	SLE0571_2-1<br>SLE0571_2-2
SLE0592	SLE0592_1-1<br>SLE0592_1-2	SLE0592_2-2	SLE0592_2-1
SLE0679	SLE0679_1-1<br>SLE0679_1-2	SLE0679_2-3	-
SLE0751	SLE0751_1-1<br>SLE0751_1-2	SLE0751_2-1	SLE0751_2-2<br>SLE0751_2-3
SLE0852	SLE0852_1-1<br>SLE0852_1-2	SLE0852_2-1	SLE0852_2-2

Table S3. Gene-based rare variant burden analyses

Locus	#variants	#variants/Kb[a]	SLE[b]	Anti-dsDNA[b]	Renal with Hypocomp-lementemia[b]
GFPT2	25	6.28	0.35	0.26	0.14
C1QTNF4	9	5.34	1	1	1
UBN2	63	4.31	0.26	0.07	0.11
BICD1	68	4.24	0.38	0.5	0.45
MICALL1	29	3.76	1	1	0.22
LRP1	25	3.76	1	1	0.57
SRRM2	37	3.37	1	1	0.29
PLD1	55	3.34	0.6	0.24	1
HMSD	5	3.04	0.71	0.03	1
GJB3	11	2.71	1	1	1
ANXA3	12	2.45	1	1	1
PRKCD	13	2.07	0.0028*	0.06	0.71
PNPLA1	11	1.67	0.04	0.02	1
DNMT3A	24	1.33	0.0075	0.0005*	0.0033*
Grouped	446	3.29	0.57	0.31	0.23

[a] Individual genes are ordered by descending rate of observed rare variants per Kb of coding exons.
[b] P-values of burden test are shown. Significant p-values at 5% FDR are highlighted with an asterisk (q=0.003)

Table S4. Minimal evidence of common variant association in European GWAS

Although none of the *de novo* mutation genes have been identified in SLE GWASs[12,13], we performed a common variant (MAF>1%) case-control analysis in 10,995 individuals of matched European ancestry using the UK10K-1000GP3 imputed data. We also performed sub-phenotype association analyses to match those used in the rare variant burden tests. Lowest *P*-value at each locus are shown. Bonferroni correction for 3,000 tests across all loci (q=1.66^{-5}).

Chr	Locus	SLE	Anti-dsDNA	Renal plus low C3
1	*GJB3*	0.0193	0.0114	0.2800
2	*DNMT3A*	0.0279	0.0063	0.0179
3	*PRKCD*	0.0600	0.0135	0.1482
3	*PLD1*	0.0026	0.0011	0.0556
4	*ANXA3*	0.0005	0.0150	0.0029
5	*GFPT2*	0.0004	0.0003	0.0433
6	*PNPLA1*	0.0214	0.0013	0.0005
7	*UBN2*	0.0121	0.0001	0.1399
11	*C1QTNF4*	0.0287	0.5997	0.0167
12	*BICD1*	0.0022	0.0010	0.0057
12	*LRP1*	0.0026	0.0041	0.0390
16	*SRRM2*	0.0286	0.0002	0.3687
18	*HMSD*	0.0293	0.05785	0.0292
22	*MICALL1*	0.0150	0.0018	0.0285

Table S5. Summary of Functions of De novo mutation gene

Data were obtained from systematic searches on GeneCards and PubMed

Gene	Gene Description	Gene Function
GJB3	gap junction protein, beta 3, 31kDa	The encoded protein is a component of gap junctions, which are composed of arrays of intercellular channels that provide a route for the diffusion of low molecular weight materials from cell to cell.
DNMT3A	DNA (cytosine-5-)-methyltransferase 3 alpha	A DNA methyltransferase that functions through de novo methylation, rather than maintenance methylation. DNMT3A and DNMT3B also carry out maintenance – correcting the errors of DNMT1. DNMT3A is essential for genetic imprinting
PRKCD	protein kinase C, delta	Protein kinase C (PKC) is a family of serine- and threonine-specific protein kinases that can be activated by calcium and the second messenger diacylglycerol. PKC family members phosphorylate a wide variety of protein targets and are known to be involved in diverse cellular signaling pathways. PKC family members also serve as major receptors for phorbol esters, a class of tumor promoters. Studies both in human and mice demonstrate that this kinase is involved in B cell signaling and in the regulation of growth, apoptosis, and differentiation of a variety of cell types. Negatively regulates B cell proliferation and also has an important function in self-antigen induced B cell tolerance induction.
ANXA3	annexin A3	Members of this calcium-dependent phospholipid-binding protein family play a role in the regulation of cellular growth and in signal transduction pathways. Inhibitor of phospholipase A2, also possesses anti-coagulant properties. Also cleaves the cyclic bond of inositol 1,2-cyclic phosphate to form inositol 1-phosphate.
GFPT2	glutamine-fructose-6-phosphate transaminase 2	Controls the flux of glucose into the hexosamine pathway. Most likely involved in regulating the availability of precursors for N- and O-linked glycosylation of proteins.
UBN2	ubinuclein 2	GO annotations related to this gene include transcription factor activity, sequence-specific DNA binding.
C1QTNF4	C1q and tumor necrosis factor related protein 4	Encodes CTRP4, which may act as a pro-inflammatory cytokine, that induces the activation of STAT3 and NF-kappa-B signaling pathway and up-regulates IL6 production. CTRP4 plays an important role in inflammation and inflammation-associated colon tumorigenesis
BICD1	bicaudal D homolog 1 (Drosophila)	Regulates coat complex coatomer protein I (COPI)-independent Golgi-endoplasmic reticulum transport by recruiting the dynein-dynactin motor complex. GO annotations related to this gene *include* transcription factor activity, sequence-specific DNA binding *and* structural constituent of cytoskeleton*.*
LRP1	low density lipoprotein receptor-related protein 1	This gene encodes a member of the low-density lipoprotein receptor family of proteins. This endocytic receptor is involved in endocytosis and phagocytosis of apoptotic cells, intracellular signaling, and lipid homeostasis.
SRRM2	serine/arginine repetitive matrix 2	Involved in pre-mRNA splicing. May function at or prior to the first catalytic step of splicing at the catalytic centre of the spliceosome. May do so by stabilizing the catalytic centre or the position of the RNA substrate (By similarity).
HMSD	histocompatibility (minor) serpin domain containing	A polymorphism in this gene may result in the expression a splice variant that encodes a minor histocompatibility antigen. This allelic splice variant of HMSD is the precursor of the histocompatibility antigen ACC-6. More generally, minor histocompatibility antigens (mHags) refer to immunogenic peptide which, when complexed with MHC, can generate an immune response after recognition by specific T-cells. The peptides are derived from polymorphic intracellular proteins, which are cleaved by normal pathways of antigen processing. The binding of these peptides to MHC class I or class II molecules and its expression on the cell surface can stimulate T-cell responses and thereby trigger graft rejection or graft-versus-host disease (GVHD) after hematopoietic stem cell transplantation from HLA-identical sibling donor. ACC-6 is presented to the cell surface by MHC HLA-B*4403. It induces cell recognition and lysis by CTL. Immunogenicity of most autosomal mHags results from single-nucleotide polymorphisms that cause amino-acid substitutions within epitopes, leading to the differential recognition of peptides between donor and recipient
PLD1	phospholipase D1, phosphatidylcholine-specific	Phosphatidylcholine-specific, membrane-associated, activated by ADP-ribosylation factor 1 by protein kinase C alpha, and Rho A, involved in inflammation, metabolic regulation, oncogenesis, neural and cardiac stimulation.
PNPLA1	patatin-like phospholipase domain containing 1	Expressed in the skin epidermal keratinocytes, and has a role in glycerophospholipid metabolism in the cutaneous barrier.
MICALL1	MICAL-like 1	On endosome membranes, may act as a downstream effector of Rab proteins recruiting cytosolic proteins to regulate membrane tubulation. May be involved in a late step of receptor-mediated endocytosis regulating for instance endocytosed-EGF receptor trafficking. Alternatively, may regulate slow endocytic recycling of endocytosed proteins back to the plasma membrane.

Table S6. Systematic search for supporting evidence for a role of *de novo* mutation gene in autoimmunity

Gene	Monogenic SLE	Functional Interactions	Immune Phenotype in Mouse Model	Rare Variant observations in autoimmunity	ImmunoBase Autoimmune Associations	Significant Candidate Gene Studies	Monogenic Disease Associations (MIM number)
MICALL1	-	-		-	-	-	-
PRKCD	Homozygous splice site variant; HomozygousG510S	C1QBP, NCF2	prone to autoimmune disease	-	UC, IBD, CD	-	Autoimmune lymphoproliferative syndrome, type III (65551)
LRP1	-	C1QA	-	T3P missense variant in Multiple Autoimmune Syndrome (MAS)	-	-	-
PNPLA1	-	-	-	-	-	-	autosomal recessive congenital ichthyosis (615024)
DNMT3A	-	-	-	-	CD	rs1550117	Tatton-Brown-Rahman syndrome (615879)
ANXA3	-	-	-	-	RA	-	-
PLD1	-	-	-	-	-	-	-
GFPT2	-	LRP1	-	-	-	-	-
UBN2	-	-	-	-	-	-	-
SRRM2	-	-	-	-	-	-	-
C1QTNF4	-	-	-	-	-	-	-
HMSD	-	-	-	-	-	-	-
BICD1	-	-	-	-	-	-	-
GJB3	-	-	Erythrokeratodermia variabilis et progressiva	-	-	-	Erythrokeratodermia variabilis et progressive (133200); Deafness (612644)

UC=Ulcerative colitis; IBD=Inflammatory bowel disease; CD=Crohn’s disease; RA=Rheumatoid Arthritis
